# Screening Novel Molecular Targets of Metformin in Breast Cancer by Proteomic Approach

**DOI:** 10.3389/fpubh.2017.00277

**Published:** 2017-10-16

**Authors:** Lobna Al-Zaidan, Rasha Abu El Ruz, Ahmed M. Malki

**Affiliations:** ^1^Biomedical Sciences Department, College of Health Sciences, Qatar University, Doha, Qatar

**Keywords:** metformin, MCF-7, cell cycle, p53, proteome

## Abstract

Metformin is a commonly prescribed antihyperglycemic drug, and has been investigated *in vivo* and *in vitro* for its effect to improve the comorbidity of diabetes and various types of cancers. Several studies investigated the therapeutic mechanisms of metformin on cancer cells, but the exact mechanism of metformin’s effect on the proteomic pathways of cancer cells is yet to be further investigated. The main objective of our research line is to discover safe and alternative therapeutic options for breast cancer, we aimed in this study to design a novel “bottom up proteomics workflow” in which proteins were first broken into peptides to reveal their identity, then the proteomes were precisely evaluated using spectrometry analysis. In our study, metformin suppressed cell proliferation and induced apoptosis in human breast carcinoma cell line MCF-7 with minimal toxicity to normal breast epithelial cells MCF-10. Metformin induced apoptosis by arresting cells in G1 phase as evaluated by flow cytometric analysis. Moreover, The G1 phase arrest for the MCF-7 has been confirmed by increased expression levels of p21 and reduction in cyclin D1 level. Additionally, metformin increased the expression levels of p53, Bax, Bad while it reduced expression levels of Akt, Bcl-2, and Mdm2. The study employed a serviceable strategy that investigates metformin-dependent changes in the proteome using a literature-derived network. The protein extracts of the treated and untreated cell lines were analyzed employing proteomic approaches; the findings conveyed a proposed mechanism of the effectual tactics of metformin on breast cancer cells. Metformin proposed an antibreast cancer effect through the examination of the proteomic pathways upon the MCF-7 and MCF-10A exposure to the drug. Our findings proposed prolific proteomic changes that revealed the therapeutic mechanisms of metformin on breast cancer cells upon their exposure. In conclusion, the reported proteomic pathways lead to increase the understanding of breast cancer prognosis and permit future studies to examine the effect of metformin on the proteomic pathways against other types of cancers. Finally, it suggests the possibility to develop further therapeutic generations of metformin with increased anticancer effect through targeting specific proteomes.

## Introduction

Breast cancer is more globally prevalent in females’ communities and classified as the second death-leading type of cancer worldwide after lung cancer (1–4). In 2015, the disease accounted for 8.8 million deaths worldwide (1). Many risk factors were found directly involved in breast cancer development such as; environmental factors, genetic makeup, reproductive factors, and behavioral or lifestyle risk factors (2). Despite the available treatment protocols, cancer patients remain at a higher risk of disease recurrence or secondary disease development, thus early detection and risk factors avoidance remain essential strategies for breast cancer prevention (3, 4). The available treatment options of chemotherapy, mastectomy, radiation, and hormonal therapy remain to be the most commonly used therapeutic strategies to increase the survival rates of breast cancer patients (5).

Metformin has been found to improve the comorbidity survival for diabetic-cancer patients, in particular breast cancer. Many *in vivo and in vitro* studies showed that metformin has an antibreast cancer therapeutic potential (6). Metformin treatment is widely prescribed for type two diabetes and has been also used off-label for the treatment of polycystic ovarian syndrome (7); however, metformin was also found to be associated with decreased incidence of various types of cancers such as breast, pancreas, hepatocellular carcinoma, colorectal, and prostate cancers (8).

Several population studies proposed that metformin reduce the incidence rate of mortality and morbidity due to cancer in patients with type two diabetes (9–11). Currie et al. revealed that diabetic patients with cancer who were treated with metformin experienced a greater survival rate in comparison with those patients who administered other antidiabetic drugs (12). A number of studies examined several mechanisms of metformin’s inhibitory effect on cancer cells, yet very limited studies investigated the prolific proteomic pathways. Algire et al. proposed that metformin has an anticancer effect by decreasing the mitochondrial reactive oxygen species production, therefore metformin decreases the oxidative stress detected by measuring several isoprostanes (13). Other studies suggested that metformin enhances the efficacy of the chemotherapeutic regimens due to its beneficial effect to overcome the chemotherapy resistance (14–18). It was reported that metformin’s radio sensitizer effect through downregulating the hyperactivity of phosphatidylinositol-3-kinase (PI3K), Akt, and mammalian target of rapamycin (mTOR) signaling pathway (PI3K-Akt-mTOR) (19).

Metformin also found to inhibit lipogeneses which is associated with cancer development (20, 21), and hyperinsulinemia has been recognized as a risk factor in cancer development of several types of cancers such as colon cancer, prostate cancer, and breast cancer (22). Metformin is also capable to induce apoptosis in several cancerous cell lines such as triple negative breast cancer, endometrial cancer, and glioma (23–25). When metformin activates adenosine monophosphate-activated protein kinase (AMPK), the tumor suppressor protein p53 will be activated and subsequently inhibit cell division and induces apoptosis (26). Additionally, metformin activates p53 and Bcl-2-associated X protein (BAX), and induces the cells to undergo apoptosis through the extracellular receptor kinase (ERK) signaling pathway (27). Another proposed mechanism of metformin, is the inhibition of angiogenesis through attenuating angiogenic stimuli in the blood *via* decreasing the levels of vascular endothelial growth factor (28). Metformin suppresses inflammation through the inhibition of several mediators such as hypoxia-inducible transcription factor-1 alpha, tumor necrosis factor alpha (TNF-α) through inhibition of mTOR signaling (29).

Over a period of 30 years, the study of proteins using mass spectrometry (MS) and molecular techniques has evolved and proteomics have been recognized as an efficient tool for research investigations (30, 31). Studying the proteome reveals the structure, function, and interaction of the proteins through comparing the resultant information in previously established databases which would help identifying novel proteins and signaling mechanisms (31, 32).

In this study, we adopted “bottom up proteomics workflow” in which proteins were first broken down into peptides by enzymatic digestion prior to MS analysis. The resulted data of individual peptides have been reconstructed using databases in order to reveal the proteins identity (33). Digested peptides were subjected to isotopic labeling which allowed the quantitative determination of proteins intensities by tandem MS (31). Isotopomeric dimethyl labeling is used to label peptide and this methodology ensured labeling peptides with labels that carry different masses which were further identified in a single MS run for comparison purposes (31, 34). Our findings proposed prolific proteomic changes that revealed the therapeutic mechanisms of metformin on breast cancer cells and normal breast cells upon their exposure.

## Materials and Methods

### Experimental Agents

1,1-Dimethylbiguanide hydrochloride; metformin NH_2_C(=NH)NHC(=NH)N(CH_3_)_2_·HCl Mw 165.62 (Fluka, Germany, cat. no. 1115-70-4). To prepare 100 mM metformin in 1 mL stock solution; 0.0165 g of metformin crystals were measured and dissolved in 1 mL media then filtered by 0.25 μm filter. The prepared solution was stored at 4°C.

### Cell Lines and Culture Conditions

The (human breast epithelial adenocarcinoma MCF-7 WT P53) cell line was purchased from the American Type Culture Collection (ATCC-HTB-22™). The human normal epithelial breast cells (MCF-10A) was purchased from the American Type Culture Collection (ATCC-CRL-10317™). MCF-7 cells were cultured in Dulbecco’s modified Eagle’s medium (DMEM) 1,000 mg/L of glucose (Sigma-Aldrich D6046) containing 10% fetal bovine serum (Hyclone, SH30070.30) and 1% penicillin/streptomycin.

MCF-10A cells were cultured in mammary epithelial cell growth medium MEGM (Lonza, CC3151) along with the additives of Lonza clonetics kit (Lonza, CC4136) containing supplement and growth factors (bovine pituitary extracts, human epidermal growth factor, hydrocortisone, and insulin) without using the gentamycin amphotericin B mix provided with the kit.

### Cell Proliferation Assay [3-(4,5-Dimethylthiazol-2-yl)-2,5-Diphenyl Tetrazolium Bromide]

Cells were seeded at a density of 10 × 10^3^/well in a 96-well plate for 24 h prior to compounded treatment. Different concentrations of the medication was added (0, 2.5, 5, 7.5, 10, 15, and 20 mM metformin) for 24 h in culture before incubating cells with [3-(4,5-dimethylthiazol-2-yl)-2,5-diphenyl tetrazolium bromide] for 4 h in a humidified 37°C and 5% CO_2_ atmosphere. MTT is reduced to purple formazan salt crystals in living cells which in turn are dissolved in dimethyl sulfoxide and spectrophotometrically quantified using ELISA reader (PerkinElmer Multilabel reader, Waltham, MA, USA) at 570 nm. The data were expressed as the percentage of absorbance as compared to untreated cells.

### Apoptosis Assay by PE Annexin V Apoptosis Detection Kit 1

Cells were seeded in a T75 flask and incubated at 37°C humidified incubator, 5% CO_2_ to reach confluency. Cells then were incubated with or without 10 mM metformin for 24 h. Cells then were harvested in an appropriate manner and 1 × 10^6^ cells were washed twice with cold phosphate-buffered saline (PBS). Cells were then re-suspended in 100 μL 1× Annexin V-binding buffer provided with the kit followed by adding 5 μL 7AAD and 5 μL PE Annexin V to each sample, gently vortexed, and incubated for 15 min in the dark. Finally, 400 μL of 1× Annexin V-binding buffer was added to each sample. Cells then were counted by BD LSRFortessa X-20 cell analyzer (Becton Dickinson, Franklin Lakes, NJ, USA) flow cytometry. Cells without treatment were used as negative controls.

### Cell Cycle Analysis of MCF-7 Cells

Cells were seeded at a density of 1 × 10^6^ cells in a T75 flasks and incubated to reach 80% confluence. The cells were treated with 10 mM metformin and harvested by trypsinization at 48 h posttreatment. 1–2 × 10^6^ cells for each condition treated and untreated cells as a negative control were washed with PBS and fixed with ice-cold 70% ethanol drop wise while vortexing to avoid clumping of the cells followed by at least 3 h incubation in 4°C. Then, the cells were washed and re-suspended in PBS containing 5 μg/mL RNase A and incubated for 30 min at room temperature. Finally 50 μg/mL Propidium iodide was added to each sample. Cell cycle analysis was performed using BD LSRFortessa X-20 cell analyzer (Becton Dickinson, Franklin Lakes, NJ, USA) flow cytometry according to the manufacturer’s protocol. BD Facs, Diva 8.0 software was used to calculate resultant DNA concentrations to determine the cell cycle phase distribution, and the results were statistically expressed as percentage of cells in the G0-G1, S, and G2-M phases.

### Morphological Appearance by Light Microscopy

MCF-7 and MCF-10A of 10 mM metformin treated for 24 h were observed for morphological changes and compared to the untreated control cells using light microscopy (ThermoFisher Scientific, advanced microscopy group, EVOS) and photographed at 4× magnification.

### TUNEL and DAPI Staining by Confocal Microscopy

TUNEL assay was performed using DeadEnd™ Fluorometric TUNEL staining for *in situ* detection of apoptotic cells by confocal microscopy. Cells were cultured in four 35 mm glass bottom microwell dishes, poly-d-lysine coated, γ-irradiated (MatTek Corporation, P35GC-0-10-C) at a density of 2 × 10^5^ cells/dish and incubated for 24 h. After treatment with 10 mM metformin for 24 h, treated and untreated control cells were fixed with 4% formaldehyde in high-performance liquid chromatography (HPLC) grade water, permeabilized with 0.2% Triton-Octyl phenol ethoxylate, equilibrated with equilibration buffer, labeled with TdT reaction mix, washed, and DAPI mounting stain was finally added. Finally, the cells were observed with inverted confocal microscope (Carl Zeiss LSM-710) and photographed at 40× magnification.

### Protein Quantification and In-Solution Digest

Protein samples were quantified using Bradford 1× dye using (PerkinElmer, Waltham, MA, USA, Multilabel Reader) machine. The protein standard curve was established by duplicate readings of different concentrations of a known protein (bovine serum albumin 2 mg/mL) in which the concentration of the samples compared to control. Samples are prepared for MS analysis adopting bottom-up proteomics workflow; proteins should be first digested into peptides by trypsinization in which we call (in-solution digest). MS analysis is performed then on an aqueous mixture of the individual peptides, and the information is then reconstructed to reveal the protein identities. Six protein samples were subjected to digestion in which three were protein of untreated control cells of three different biological replicates and three were protein of 10 mM metformin-treated cells of three different biological replicates. 100 μg protein of each sample was digested separately. 1.5 μL of 10 mM dithiothreitol (DTT) reducing agent was added to each of the six samples and incubated for 30 min. DTT was added for the purpose of converting cysteine’s disulfide bond into cysteine free sulfhydryl groups. Then, 1.7 μL of 55 mM iodoacetamide (IAA) was added to each sample followed by 30 min incubation in the dark. IAA is an alkylating agent that transforms the free sulfhydryl group of cysteine residues into S-carboxyamidomethyl-cysteine which prevents the formation of disulfide bonds. This is important for maximum cleavage by trypsin. Samples were then subjected to protein precipitation using chloroform and methanol. Protein pellets were dried and 100 μL of 100 mM triethylammonium bicarbonate buffer 1.0 M was added to each sample (33). Finally 4 μL of 0.4 μg/μL trypsin in 50 mM ABC (Trypsin, frozen) was added to each sample and samples were incubated overnight in 37°C shaker machine (Eppendorf, Hamburg, Germany, Thermomixer comfort). Trypsin cleaves C-terminal of lysine and arginine residues (35).

### Multiplex Peptide Stable Isotope Dimethyl Labeling

In this method, the N-terminus of the proteins/peptides and ε-amino group of lysine are converted to dimethylamines through reductive amination. The previously digested samples were subjected to chemical labeling, in which, untreated samples were labeled with light dimethyl labeling compounds while the treated samples were labeled with heavy dimethyl labeling compounds. For light labeling, 16 μL of 4% (v/v) formaldehyde solution CH_2_O prepared in HPLC grade water was added to each sample of the light labeling proteins (untreated control cells). Samples were mixed and spin down then 16 μL of 0.6 M NaBH_3_CN sodium cyanoborohydride prepared in HPLC grade water was added to each sample, mixed, and spin down. For heavy labeled samples, 16 μL of 4% (v/v) formaldehyde ^13^C-d_2_ solution (^13^CD_2_O) marked as (F-^13^C-d_2_) prepared in Deuterium water D_2_O was added to each sample, mixed, and solution was spin down. Then, 16 μL of 0.6 M of NaBD_3_CN sodium cyanoborodeutride prepared in deuterium water D_2_O, was added to each sample, mixed, and spin down. All the six samples were then incubated at room temperature with gentle mixing using a thermos mixer. To quench the labeling reaction, 64 μL of 1% (v/v) ammonia solution in HPLC grade water was added to each sample, then mixed and spun down. Then, 32 μL of 5% (v/v) formic acid in HPLC grade water, was added to each of six samples, mixed and spun down (34, 36). Finally, each light and heavy labeled samples of the same biological replicate were mixed yielding three samples. The three samples were then subjected to reversed phase resin after labeling *via* Oligo R_3_ reversed phase packing beads and POROS 50 R_2_ beads, 1:1 mixture resuspended in acetonitrile ACN. Self-made stop and go extraction tips (stage tips) with one layers of C18 (solid phase extraction disk, C18 octadecyl) disks in a P200 pipette tip were prepared and filled up to the half with R_2_/R_3_ beads suspension mixture. ACN was then discarded, and beads were washed with 200 μL 0.1% trifluoroacitic acid (TFA). Protein samples were then loaded in to the tips, flow through was discarded, and tips were washed with 1% TFA.

All the samples were then preserved in −80°C for subsequent steps of samples preparation for MS analysis.

### Peptide Fractionation by In-Solution Isoelectric Focusing

Three samples were ready for fractionation as mentioned previously. Each sample contains 200 μg of peptides digest. Samples were then brought to 360 μL using HPLC water, then four volumes of OFFGEL running buffer stock solution were added to each sample. OFFGEL running buffer stock solution was prepared by mixing 60 μL of the OFFGEL buffer and 300 μL of 50% glycerol brought up to 50 mL HPLC water. Three IPG frozen strips pH: 3–10 were placed in the IPG plastic tray and plastic frame cups then were placed on top of the IPG strips. IPG strips were rehydrated using 40 μL rehydration solution added to each cup. Rehydration solution was prepared by mixing 4:1 OFFGEL running buffer stock and HPLC water. Swirling strips were then incubated for 15 min. Wet electrode pads were then placed on each edge of the IPG strips. 150 μL of each sample was then loaded into each cup for all 12 cups and cups then were sealed with a silicon lid. Mineral oil was added at the anode and cathode ends and then the electrodes were placed on the IPG strips and connected to the instrument (Agilent, Santa Clara, CA, USA, G3100A, OFFGEL fractionator) where fractionation was performed for 24 h (37, 38). The fractions were then harvested and stage tipping protocol was performed.

### Stage Tipping of Peptide Fractions

The analysis of peptides by liquid chromatography (LC)/MS/MS liquid chromatography-tandem MS requires cleaning, concentrating, enriching, and prefractionating of samples. This is achieved by self-made stop and go extraction tips (stage tips). These are prepared by packing three layers of C18 (solid-phase extraction disks in a P200 pipette tip). For each sample, 12 tips were prepared; one for each fraction of the 12 fractions prepared as mentioned previously. Stage tips were then reactivated by washing with 200 μL methanol that enhances the C18 chains to interact with hydrophobic peptides yielding peptide/resin interaction. Tips then were re-equilibrated twice using 200 μL of buffer A* that promotes hydrophobic interaction in an aqueous environment and ion pairing agent TFA. Peptides were then loaded, and finally tips were washed with 200 μL buffer A to desalt the resin bond peptides. Tips were then washed with 20 μL of buffer A to rehydrate dried resin-bond peptides and the flow-through was discarded. OFFGEL Fractions were washed with 100 μL buffer A* to reduce the amount of ampholites in the samples. Buffer A* was discarded before adding buffer B60 which was then collected. Buffer B60 eluted the peptides from the tips yielding pooled elutes that were dried by speed vac to reduce sample acidity using (Eppendorf, Hamburg, Germany, concentrator plus). Finally, buffer A was added to each dried fraction of the three samples before analyzing by LC–MS/MS.

### Mass Spectrometry

Each fraction was individually submitted to analysis by nano-LC coupled to MS; the analytical platform consisting of an EASY nLC-II interfaced to a Q Exactive mass spectrometer MS (Thermo Scientific).

Chromatography conditions were defined as follows: water with 0.5% acetic acid for mobile phase A; water: acetonitrile, 20:80 volume ratio, with 0.5% acetic acid for mobile phase B; flow-rate of 250 nL/min; injection volume of 6.0 μL and a maximal loading pressure of 280 bar. LC separation was done on 20 cm long in-house packed emitter columns (ReproSil-Pur 120 C18-AQ 3 um diameter beads, Dr. Maisch GmbH) using a gradient ranging from 5 to 30% mobile phase B over 90 min, followed by a 25 min wash and column re-equilibration cycle. Data-dependent mass spectrometric acquisition employed a classical top 10 method. Briefly, the 10 most intense ions, excluding unassigned charge states and singly charged ions, detected in the preceding full scan were isolated (3 Th isolation width) and fragmented using higher energy collisional dissociation (HCD) (normalized collision energy 25). Precursor scans (MS1 level) were acquired at a resolution of 70,000 (*m*/*z* 300) and an AGC target value of 3,000,000 charges (maximum ion injection time 20 ms). Fragmentation spectra (MS2 level) were acquired at a resolution of 17,500 (*m*/*z* 300) and an AGC target value of 100,000 charges (maximum ion injection time 120 ms) and a fixed lower mass-to-charge cutoff of 100. All scan events were recorded in profile mode, a dynamic exclusion list of 25 s was employed and both the exclude isotopes and peptide match functionalities were activated.

### Data Analysis

Max Quant was used to process the raw mass spectra and generate protein group intensities and normalized ratios. A statistical analysis of these normalized ratios was performed using the in-house R package comics. MS data were analyzed by MaxQuant suite of algorithms version 1.5.2.8, using a Homo Sapiens database downloaded from UniprotKB on the October 12, 2015, and comprising 48,460 reviewed entries (canonical and isoforms). The following default search parameters were employed; first search mass accuracy tolerance 20 ppm, main search mass accuracy tolerance 4.5 ppm, FTMS MS/MS tolerance 20 ppm, minimum peptide length of seven amino acids, peptide spectrum match FDR and protein FDR both set to 0.01 as calculated by the revert database approach. Protein quantification was based on a minimum of two ratio counts, originating from unique or razor peptides only. Additionally, unless explicitly stated otherwise, other parameters of the data analysis were not changed from their MaxQuant 1.5.2.8 default value.

Multiplicity was set to 2 with DimethNter0/DimethLys0 and DimethNter8/DimethLys8 for light and heavy labels, respectively, as required by dimethyl labeling approach. The search was conducted using Trypsin/P enzyme specificity, allowing for maximum two missed cleavages, N-terminal acetylation and methionine oxidation as variable modifications and cysteine carbamidomethylation as fixed modification. Both re-quantify and match between runs (match time: 2 min, alignment time window: 20 min) functionalities were enabled and MaxQuant analysis was performed on the combined 36 LC–MS runs using condition and replicate number for sample naming convention in the maxquant experimental design template file. The proteinGroups.txt file produced by Max Quant was loaded into R. After that, features tagged as “Reverse” or “Potential Contaminants” (both columns in the protein groups table) were filtered out. The filtered normalized ratios were then log2 transformed, in order to achieve a symmetric and approximately normal distribution. A subsequent quantile normalization was performed prior to statistical analysis, in order to make the different samples properly comparable to each other.

## Results

### Metformin Reduced Proliferation and Induced Apoptosis in MCF-7 and MCF-10A Cells

**Figure 1:**
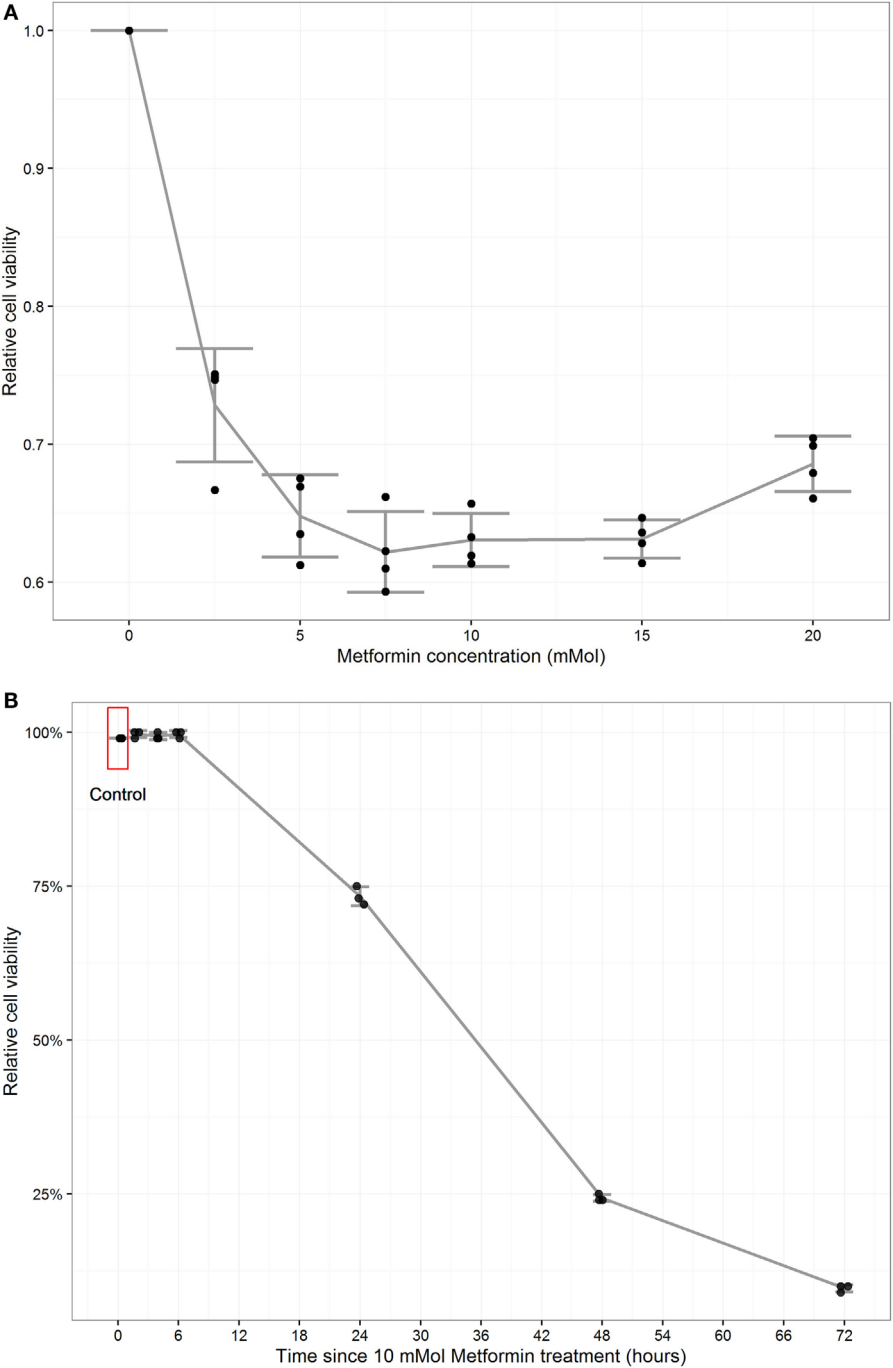

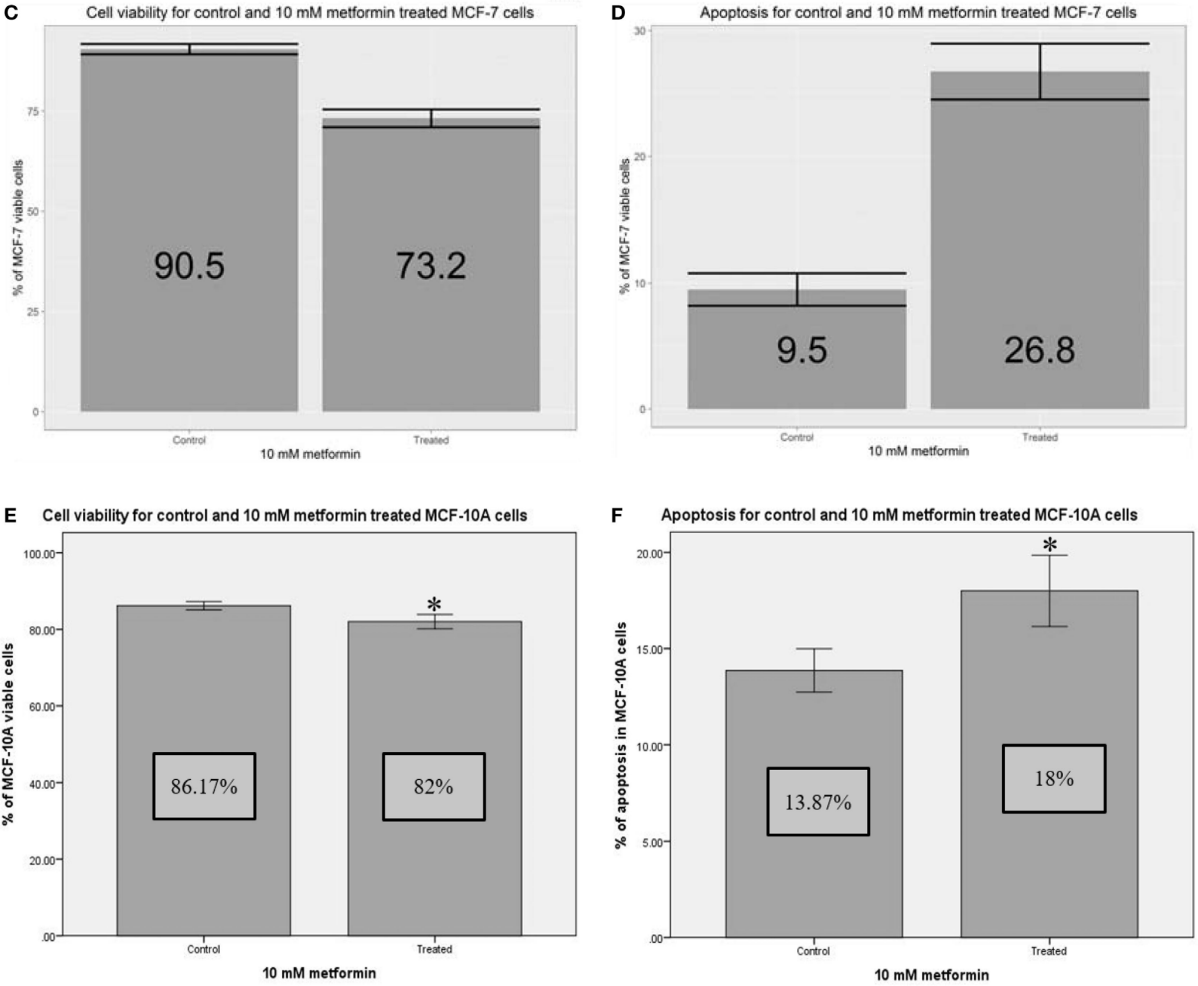
Metformin reduced proliferation and induced apoptosis in MCF-7 and MCF-10A. Cell proliferation assay (MTT) was performed to detect the percentages of living cells in MCF-7 cells. Each data point was an average of four readings presented as mean ± SD. A (dose–response curve) was constructed where 10 mM metformin concentration was chosen for subsequent experiments. Cell counting by trypan blue was performed to detect cell viability percentage. Each data point was an average of three readings presented as mean ± SD **(A)**. A time course curve was constructed from which 24 h posttreatment time point for subsequent experiments **(B)**. Cell viability and Apoptosis induction in MCF-7 cells treated with metformin: Statistical graphs show the% of viability and apoptosis in 10 mM metformin-treated cells compared to untreated MCF-10 A control cells. *P* value = 0.0004 (paired *T*-test), error bars ± 1SD. *n* = 4 **(C,D)**. Cell viability and apoptosis induction in MCF-7 cells treated with metformin, Statistical graph shows the % of viability and apoptosis in MCF-7 cells treated with 10 mM metformin in comparison with non-treated controls **(E,F)**.

Initially, we mimicked the normal physiological concentration of glucose in non-diabetic population by decreasing the glucose concentration to match with *in vivo*. To detect the effect of glucose lowering on cell viability, MCF-7 cell were cultured in two different conditions; high glucose concentration of 24 mmol/L and low glucose concentration of 6 mmol/L media for comparison. The percentage of cell viability was determined by flow cytometry using PE Annexin/7AAD apoptosis assay. Our data showed that lowering the glucose concentration did not affect cell viability of cells cultured in low glucose media (93% viable cells) in comparison to the viability of cells cultured in high glucose media (92.3% viable cells) (data not shown). Cell proliferation assay was performed to determine cell viability in MCF-7 using MTT assay. Different concentrations of metformin treatment (0, 2.5, 5, 7.5, 10, 15, and 20 mM metformin) was used and dose response curve was constructed of which we chose the concentration used in subsequent experiments. The increment of the treatment dose was correlated with reduction in viability however; concentrations above 10 mM did not decrease the viability further where it plateaued (Figure 1A). The cell proliferation assay indicated that 10 mM of the treatment lowered the viability of MCF-7 approximately 37% compared to the non-treated controls.

To determine the optimum time point for metformin application apoptosis measured by trypan blue counting for viability percentage and a time course curve was constructed. 10 mM metformin was added to cells in different time points (2, 4, 6, 24, 48, and 72 h). It was found that in 2, 4, and 6 h treatments did not induce cell apoptosis while in 24 h apoptosis was estimated by 27%. Cell viability dropped to less than 25% in 48 h and 10% in 72 h (Figure 1B). Apoptosis assay was performed on treated MCF-10A cells which revealed a minimal toxicity of 4% increase in apoptosis percentage compared to untreated controls (Figures 1C,D). The induction of apoptosis in treated MCF-7 was confirmed by the addition of 10 mM metformin for 24 h increased apoptosis up to 27% compared to 9.5% in untreated control cells (Figures 1E,F).

### Induction of Apoptosis in MCF-7 and MCF-10A by Metformin and Its Impact on Cell Cycle

**Figure 2:**
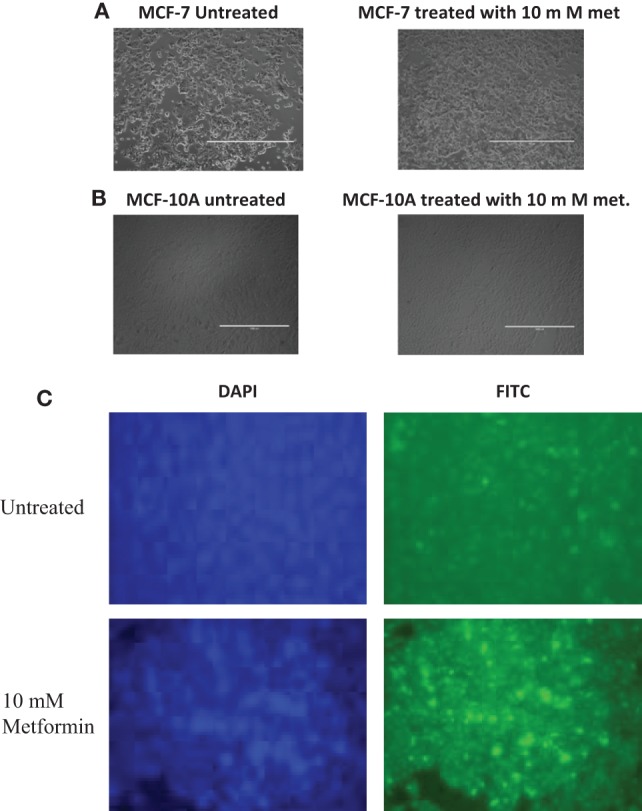

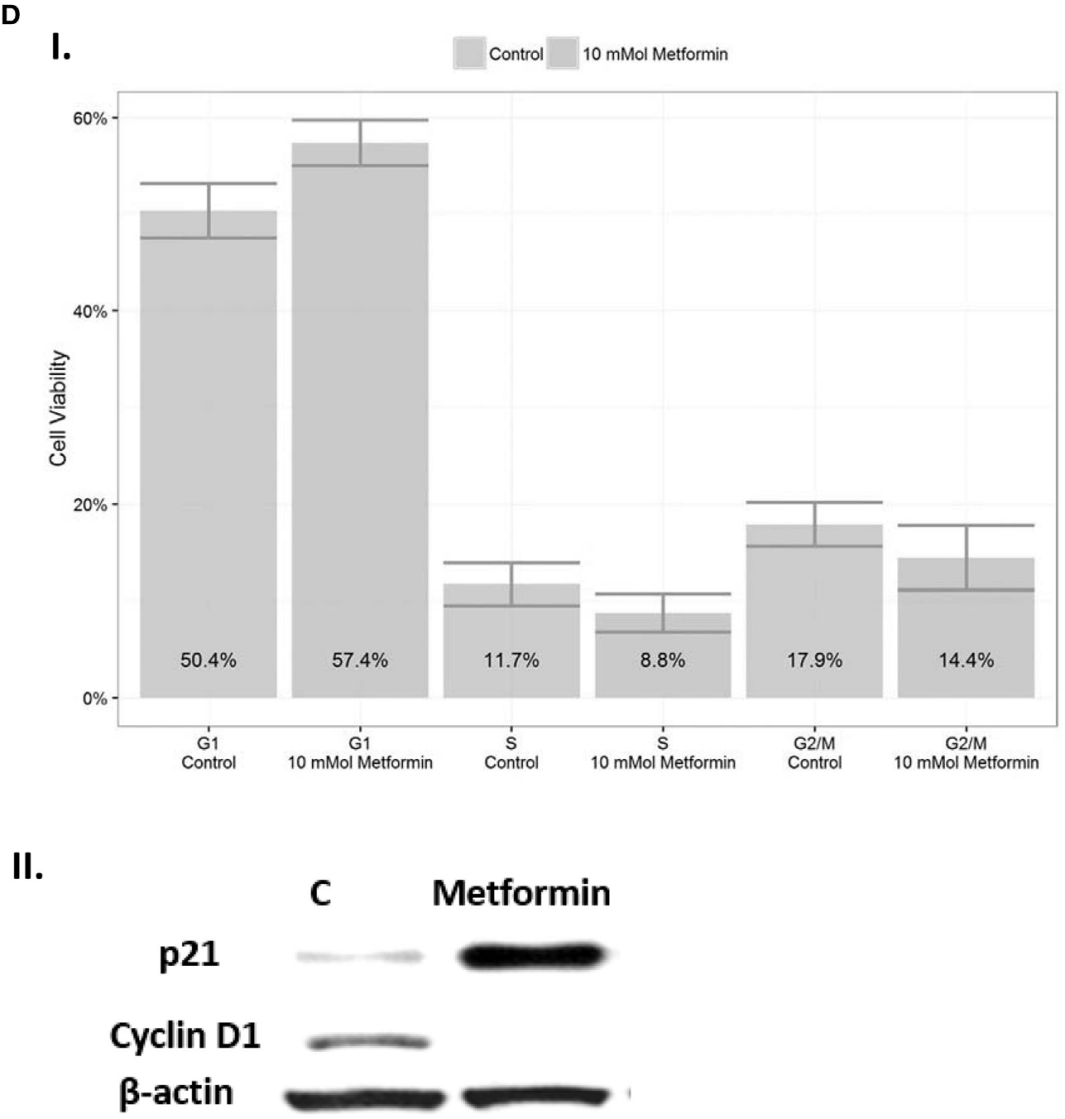
Impact of metformin on apoptosis and cell cycle checkpoints. Cells were treated for 24 h with 10 mM metformin. Untreated cells were used as controls. Light microscopy images of MCF-7 cells photographed with 4× magnification showing the morphology of apoptotic cells in the treated group compared to untreated controls indicating that metformin has an anticancer effect inducing apoptosis **(A)**. Light microscopy images of MCF-10A cells photographed with 4× magnification showing the morphology of apoptotic cells in the treated group compared to untreated controls indicating that metformin has a slight effect on normal cells compared to the cancerous cells **(B)**. Effect of metformin on morphological appearance of MCF-7 cells. TUNEL assay, images photographed with 40× magnification showing increased number of apoptotic MCF-7 cells after treatment compared to untreated control cells **(C)**. The impact of metformin on MCF-7 cell cycle. Cells were treated with 10 mM metformin and incubated for 48 h. The percentages of cell cycle phases (G1, S, and G2/M) were determined. Untreated control cells were incubated and harvested for the same time interval. Each data point present the mean of three independent experiment values and data were expressed as mean in percentage with ±SD, Western blot analysis showed variations in p21 and cyclin D1 levels **(D)**.

MCF-7 and MCF-10A cells of untreated and 10 mM metformin-treated cells were incubated for 24 h and images by light microscope were obtained. The morphology of the treated cells in comparison to non-treated controls revealed apoptotic features such as shrinkage and blebbing of the cells with lower degree in treated MCF-10A compared to the treated MCF-7 cells (Figures 2A,B). Microscopic slides of treated vs. untreated MCF-7 cells were prepared using TUNEL assay to be examined by confocal microscope to ascertain the induction of apoptosis. Slides revealed an increase in the number of TUNEL positive cells confirming DNA fragmentation and nuclear condensation in 10 mM treated cells in comparison to untreated controls (Figure 2C).

To study the effect of metformin on cell cycle check points in MCF-7-treated cells, cell cycle analysis was performed using flow cytometry and PI staining. Cells were treated with 10 mM metformin for 48 h before manipulating and the percentages of cell cycle phases (G1-S-G2/M) were determined. Results showed an increase in the percentage of treated cells in G1 phase of (57.4%) compared to (50.4%) in the control group, while the percentage of cells in G2 phase was reduced to (14.4%) compared to (18%) in untreated control cells (Figure 2D). Results were further confirmed by increased expressions levels of p21 and reduction in cyclin D1 level.

### Deep Proteomics Analysis of the Effect of Metformin on MCF-7 and MCF10A Cells

#### The Experimental System

**Figure 3:**
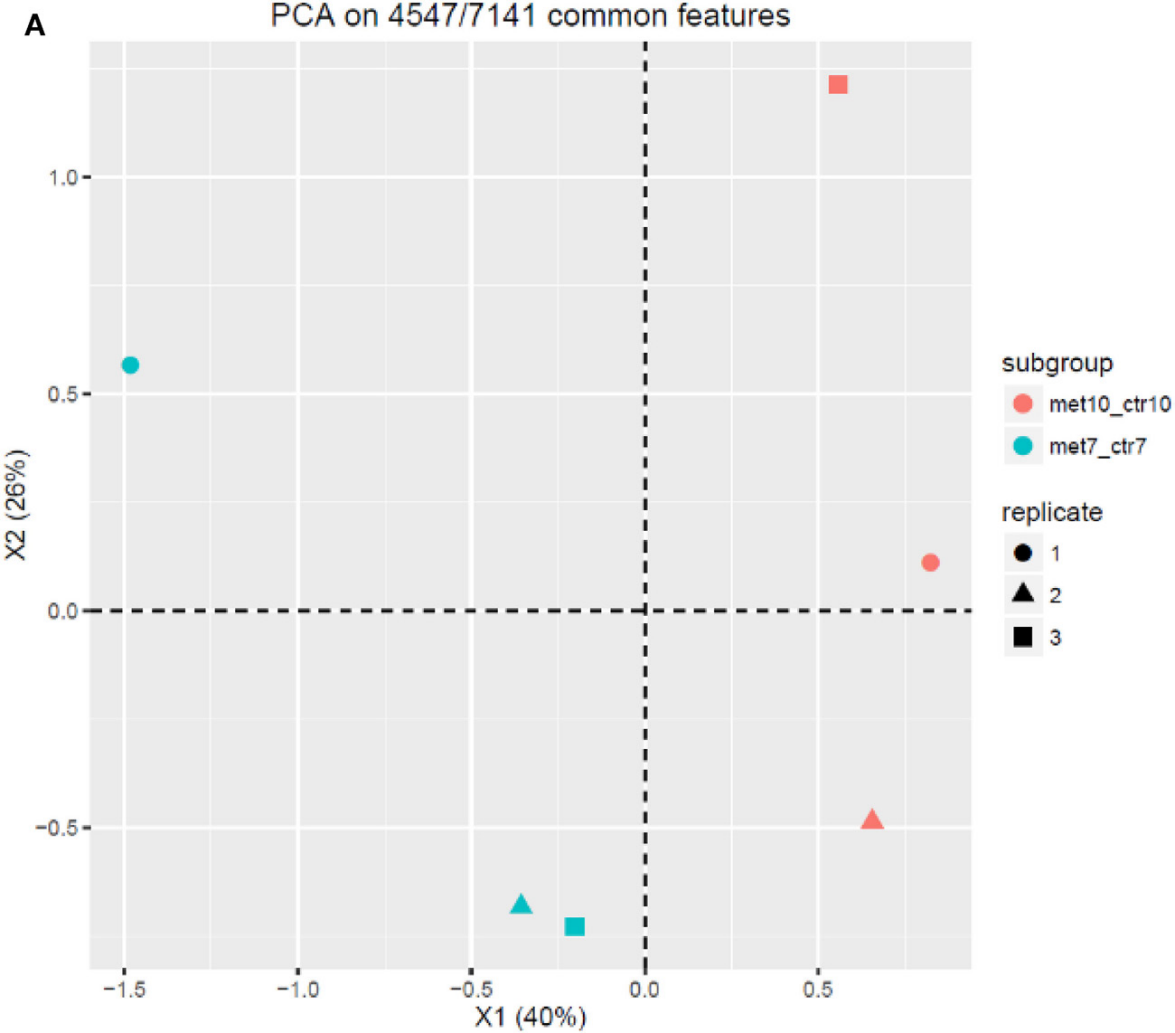

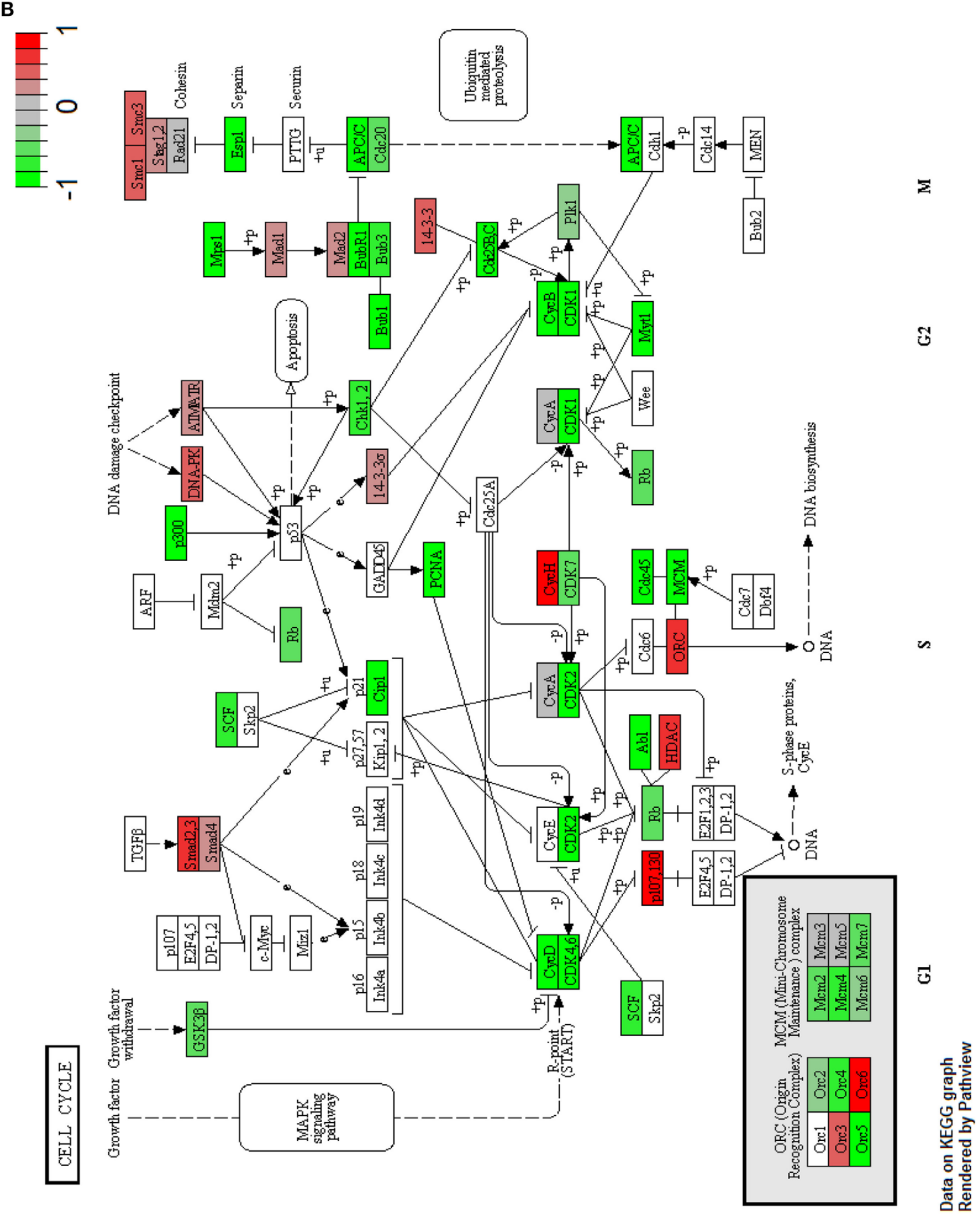

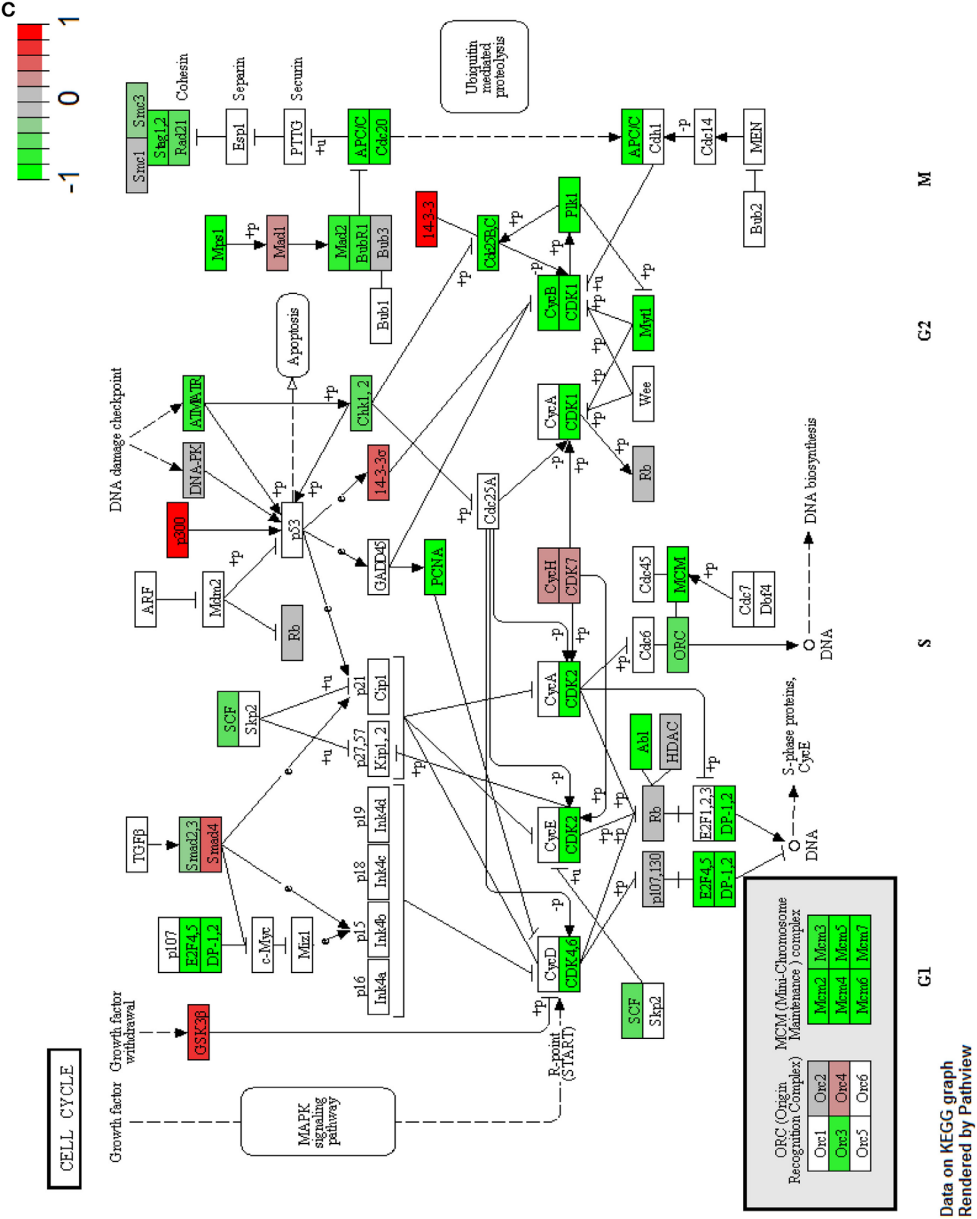

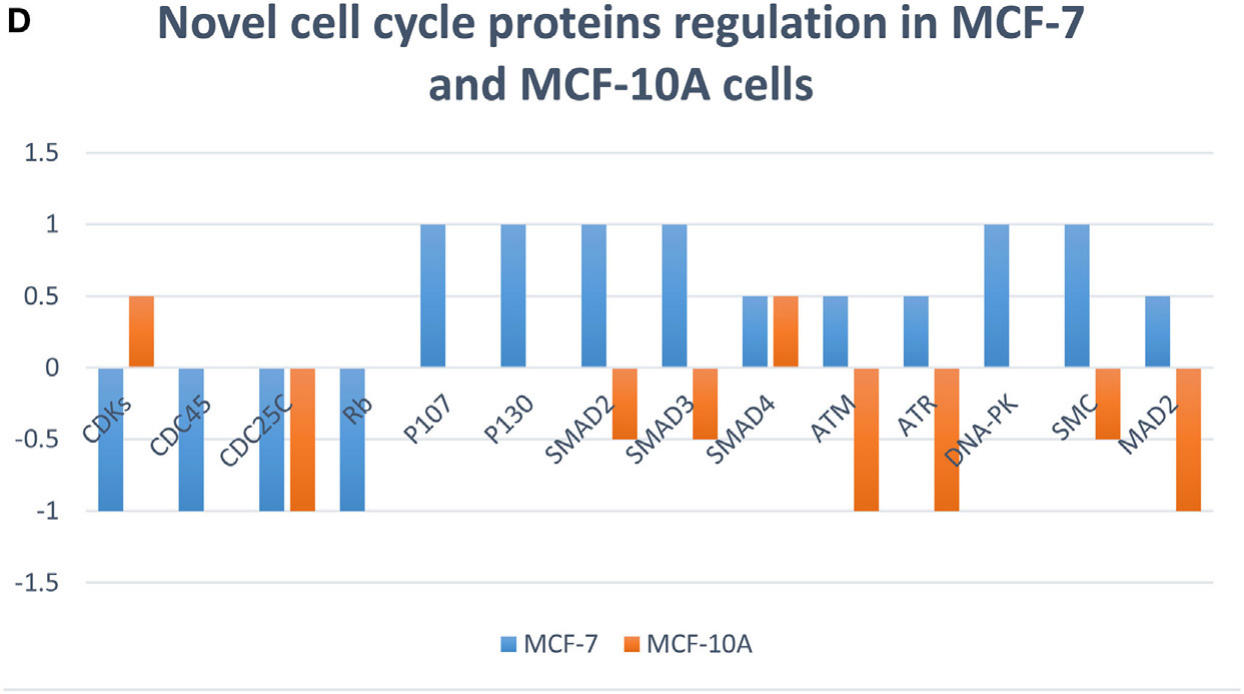
Proteomic profiling and analysis. Principle component analysis of the overall proteomics profile showing the expected effect of metformin treatment comparing deferentially expressed proteins in MCF-7 and MCF-10A cells with replicates showing similar behavior **(A)**. The cell cycle of MCF-7 cells with highlighted up- and downregulated proteins after treatment with 10 mM metformin for 24 h in comparison with untreated control **(B)**. The cell cycle of MCF-10A cells with highlighted up- and downregulated proteins after treatment with 10 mM metformin for 24 h in comparison with untreated control **(C)**. Statistical graph of novel cell cycle proteins expression in MCF-7 compared to MCF-10A cells **(D)**.

In our proteomics analysis, we analyzed protein extracts of cells treated in the same manner we followed in our previous cellular analysis. To measure protein expression levels, we used multiplex peptide stable isotope dimethyl labeling both light labeling (untreated MCF-7 and MCF-10A cells), and heavy labeling (treated MCF-7 and MCF-10A cells). Samples of treated and untreated cells of the same biological replicate were mixed and analyzed for comparison analysis done in triplicate. Each sample was fractionated by in-solution isoelectric focusing (using the Agilent 3100 OFFGEL fractionator) to 12 fractions. Each fraction was individually submitted to analysis by nano-LC coupled to MS; the analytical platform consisting of an EASY nLC-II interfaced to a Q Exactive mass spectrometer MS (Thermo Scientific). Max Quant was used to process the raw mass spectra and generate protein group intensities and normalized ratios. A statistical analysis of these normalized ratios was performed using the in-house R package comics. This method quantified the concentration of 6,295 proteins in MCF-7 cells with 2,572 Proteins differentially expressed in treated cells in comparison to untreated cells, and 6,039 Proteins in MCF-10A cells with 1,751 Proteins differentially expressed in treated versus untreated cells (Figure 3A). We compared the resultant information to KEGG PATHWAYS database and pathways with highlighted up- and downregulated components were constructed. We chose to study the pathways that revealed the antitumorigenic effect of the medication on cell viability, survival, proliferation, and apoptosis, therefore we chose to study the cell cycle, insulin receptor, and apoptosis signaling pathways (Figures 3B,C). Metformin was known to effect cellular metabolism and processes *via* its activation of AMPK (39, 40), so we thought to study its signaling and further explained the probabilities of metformin action in cellular metabolism (Figure 3D).

#### Principal Component Analysis

Principle component analysis is to study the expected effect of the metformin on the overall proteomics profile and to illustrate that experimental replicates show similar behavior. The PCA in this report was performed using mpm (41, 42). We found 4,547 proteins out of 7,141 proteins were common among all experimental groups. Each plot or each sample was thought to be as a point of the 4,547 common features in to a limited number of principle components values and each sample of the samples were colored according to cell type (MCF-7 and MCF-10A).

The data set revealed that the sample biplot distribution over the X1 and X2 principle components were significantly variable between the two groups revealing distinct effect of the medication on both cell lines and so giving a very good summary of the overall omics profile. This shows a general distinction and the resultant information may thus be valid (Figure 3A).

#### Impact of Metformin on Cell Cycle

Accumulating experimental evidence has revealed an antiproliferative effect of metformin on mitotic cell cycle in eukaryotes (27, 43, 44). The data showed decreased expression of CDKs (CDK1, CDK2, CDK4, CDK6, and CDK7) after treating MCF-7 cells with 10 mM metformin, while in MCF-10A, CDK7 and cyclin H subunit were upregulated posttreatment with metformin. Cyclin H and CDK7 forms CAK complex that is able to activate CDK2 (45), CDK1, CDK4, and CDK6 by threonine phosphorylation (46), thus regulating cell cycle progression. The transcription factor E2F is one of the downstream targets of CDKs which is regulated by Rb (47). In MCF-7 cells Rb was downregulated while in MCF-10A it remained unchanged in comparison to the untreated controls, CDKs under-phosphorylate (activate) Rb resulting in its subsequent interaction and repression of E2F1 transcriptional activity, resulting in proliferation inhibition *via* cell cycle arrest (46) which might be the case in MCF-10A. On the other hand Rb1 was found to repress the transcriptional activity of cell cycle genes through recruiting a histone deacetylase (HDAC) complex, resulting in regulating chromatin structure (45, 48). HDAC is upregulated in treated MCF-7 cells and unchanged in treated MCF-10A cells. The Rb-related proteins p107 and p130 are implicated in cell cycle regulation in which p107 appears to share many structural similarities with Rb in the repressor motif and results in growth suppression; thus it appears to have the ability to arrest cell cycle in G1 phase (48) (Figures 3B,C).

P130 a member of the Rb family that acts as a tumor suppressor is critical for telomere length control *via* forming a complex with Rad-50 to block telomerase-independent telomere lengthening (49). The p107 and p130 are overexpressed in treated MCF-7 while they remain unchanged in MCF-10A-treated cells. Cyclin-dependent kinase inhibitors (CKIs) negatively regulate CDKs activity and result in the inhibition of cell cycle progression (47). In MCF-7-treated cells the CKI p21Cip1 is downregulated. SMAD family is a signal transducers and transcriptional modulators that regulates several signaling pathways such as cell growth, differentiation, proliferation, and apoptosis. Smad2 and Smad3 are recruited to the TGFß receptor and phosphorylated, thus inducing their association with family member Smad4 which is important to translocate the protein to the nucleus forming a transcription repressor complex (45). The Smad family proteins were found to be upregulated in treated MCF-7 while Smad2 and Smad3 are downregulated in treated MCF-10A therefore disrupting the complex formation and transcription repressing processes (Figure 3D).

In response to DNA damage, a number of Checkpoint kinases are activated resulting in G1 and G2 checkpoints and subsequent arrest in cell cycle. ATM kinase activates Chk2 while ATR kinase activates Chk1 by phosphorylation which in turn activates the transcriptional activity of p53 tumor suppressor protein resulting in G1 and G2 arrest (47). In MCF-7-treated cells ATM and ATR are upregulated while downregulated in treated MCF-10A cells. DNA-PK is another protein responsible for DNA repair where it acts as a molecular sensor for DNA damage. It exerts its effect on DNA repair by phosphorylating and activating the P53 tumor suppressor protein (45). In MCF-7-treated cells the protein DNA-PK is increased while in MCF-10A-treated cells the expression of the protein remains unchanged compared to the untreated controls.

In M phase, there are several proteins responsible for chromosome maintenance and DNA repair such as Smc protein which maintains chromosomes and Mad2 protein to ensure proper alignment of chromosomes on the spindle during mitosis (45), which are all upregulated in MCF-7-treated cells but are opposed in MCF-10A-treated cells. A number of CDCs were downregulated in MCF-7-treated cells such as CDC45 which is responsible for DNA replication, as well as CDC25C which is thought to suppress P53 induced growth arrest (45). Finally, ORC1, ORC2, ORC3, ORC4, ORC5, and ORC6 subunits aside with MCM2, MCM3, MCM4, MCM5, MCM6, and MCM7 complexes are responsible for genome replication in which they vary during the cell cycle phases (45).

### Impact of Metformin on Survival Signals

The insulin signaling pathway is considered as one of the survival pathways that is initiated *via* growth factor ligand formation resulting in subsequent cellular processes such as growth, morphology, motility, survival, and proliferation (45). Here, we decided to study the insulin receptor pathway to reveal the anticancer effect of metformin on cell survival.

When Insulin binds to its receptor, it forms a ligand resulting in tyrosine phosphorylation of (IRS). Upon IRS phosphorylation a subsequent series of activation is initiated through the activation of the regulatory subunit PI3K that activates (PDK1) which in turn causes Akt activation. When Akt is activated, it phosphorylates and activates mTOR which in turn activates protein synthesis *via* the activation of eIF4eukaryotic translation initiation factor (4F) complex and p70S6K which is a member of the ribosomal S6 kinase (45, 50).

**Figure 4:**
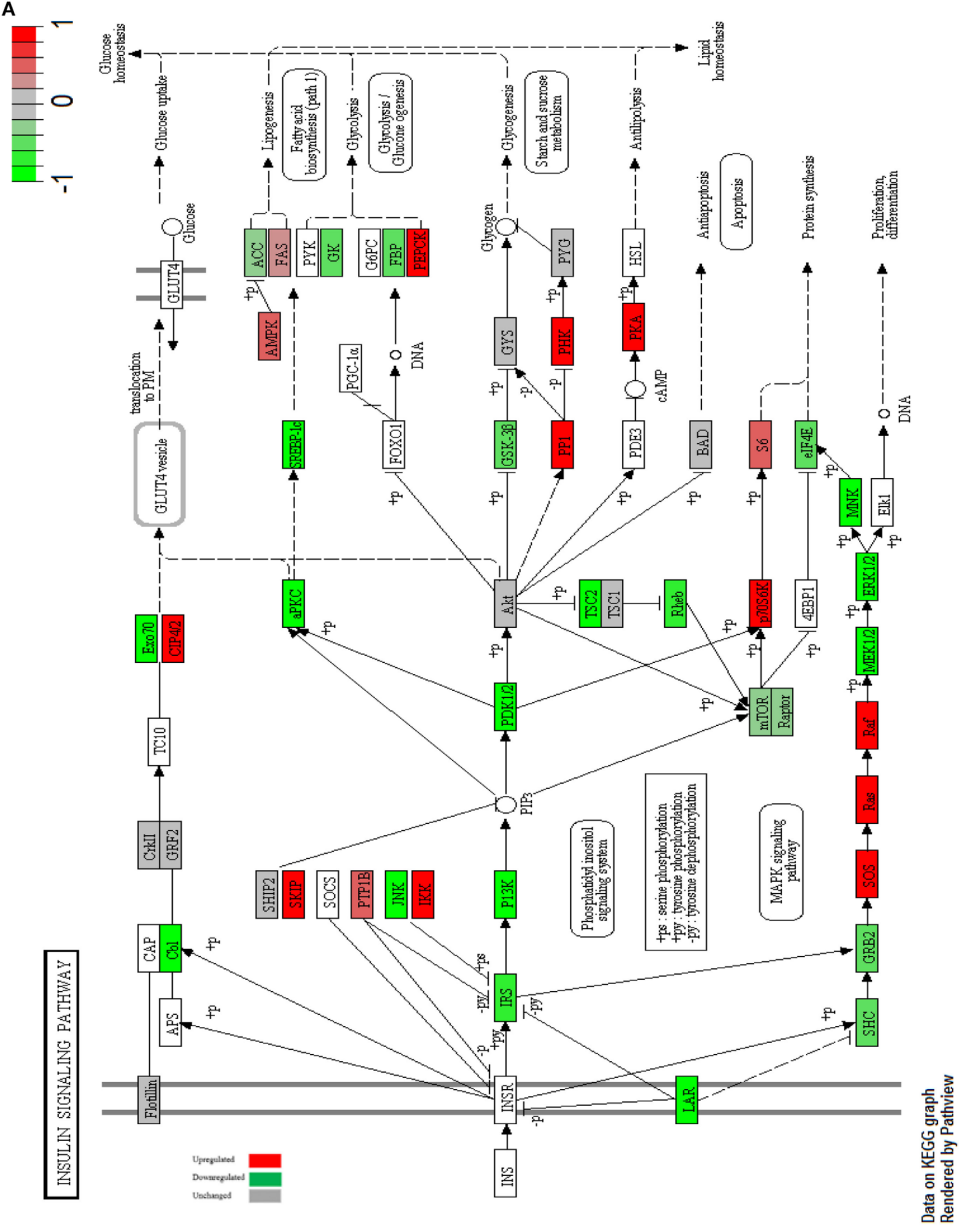

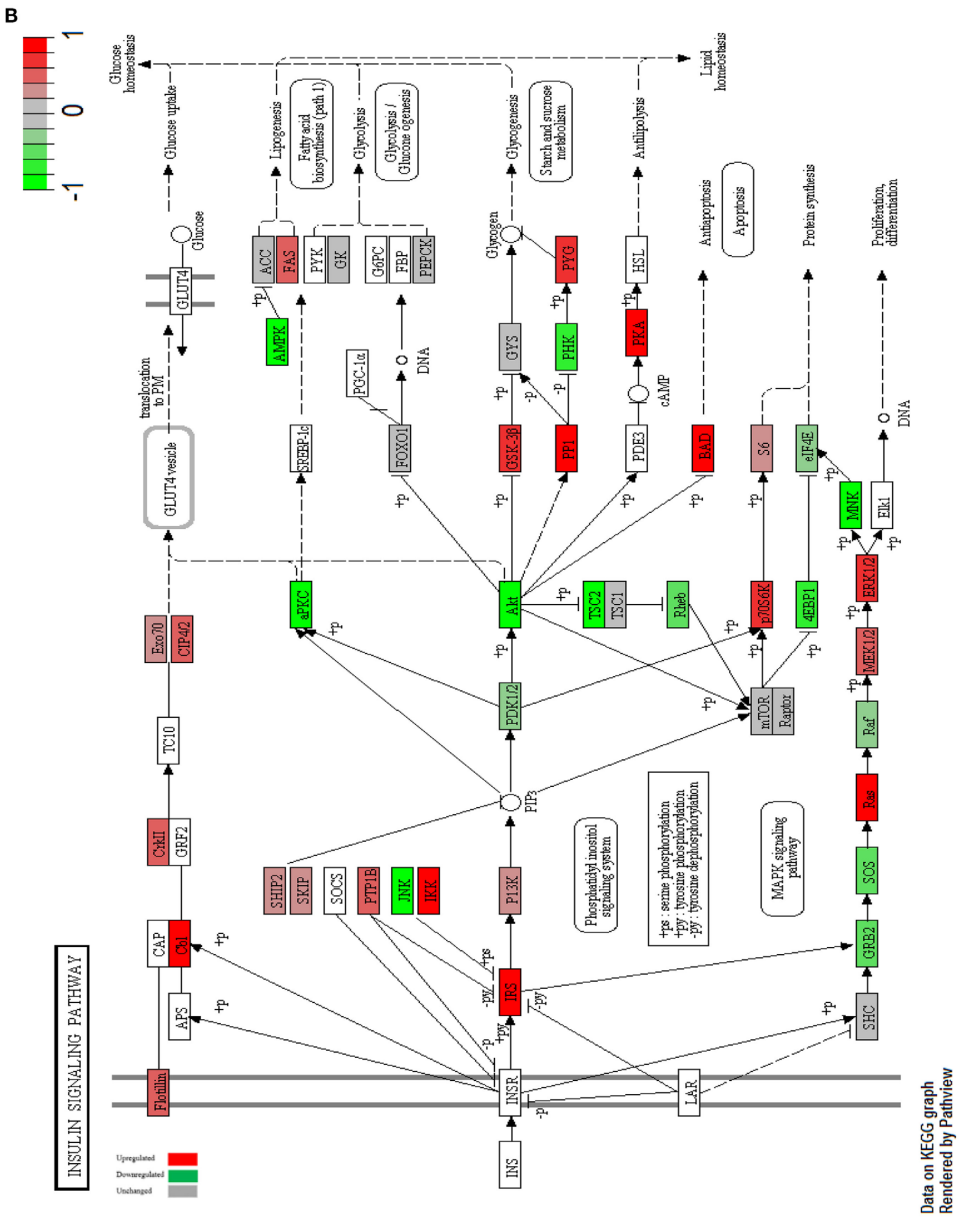
Impact of metformin on insulin receptor signaling for MCF-7 and MCF-10A. Insulin receptor signaling of MCF-7 cells with highlighted up- and downregulated proteins after treatment with 10 mM metformin for 24 h in comparison with untreated control **(A)**. Insulin receptor signaling of MCF-10A cells with highlighted up- and downregulated proteins after treatment with 10 mM metformin for 24 h in comparison with untreated control **(B)**.

mTOR is a key regulator of several cellular processes including cell metabolism, growth and survival in response to hormones, growth factors, nutrients, energy and stress signals (45), it was downregulated in MCF-7 cells after metformin treatment leading to decreased protein synthesis (Figure 4A), while in MCF-10A, the expression of mTOR did not change posttreatment. The PI3K/Akt signaling pathway is over all downregulated in MCF-7 cells (Figure 4B).

Glucose transporter 4 vesicles were translocated from their intracellular pool to the plasma membrane *via* the activation of Akt. Translocation of GLUT 4 allow cellular uptake of glucose (50). Our data revealed another type of glucose transporter which is GLUT1 that was upregulated in both cell lines post treatment which facilitates cellular glucose uptake (51). INSR insulin receptor phosphorylates Cbl which in turn activates CAP/Cbl/TC10 signal transduction pathway that consequently causes the translocation of GLUT4 protein to the plasma membrane (50). This pathway is downregulated in MCF-7 cells while upregulated in MCF-10A cells in comparison to untreated controls. IRS also results in the activation of GRB2 that activates SOS, RAS, RAF, and MEK cascade.

This transduction pathway initiates a mitogenic response through the activation of the protein kinase (MAPK or ERK) that facilitates subsequent transcriptional activity (50). The downstream signaling *via* MAPK pathway is inhibited through the downregulation of MEK1/2 and ERK1/2 in MCF-7 cells which is opposed in MCF-10A where MEK and ERK were upregulated compared to untreated control cells.

### Impact of Metformin on Apoptotic Signaling Pathways in MCF-7 Cells

**Figure 5:**
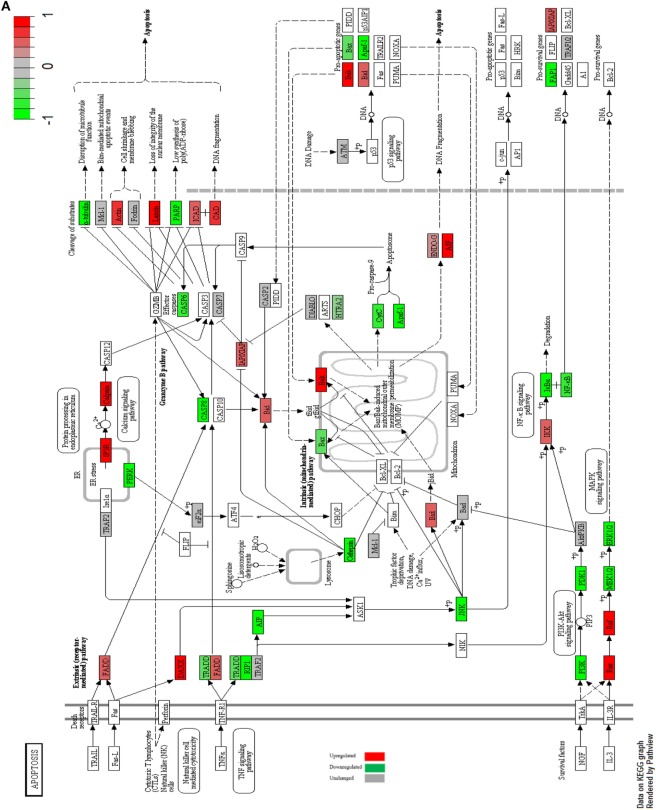

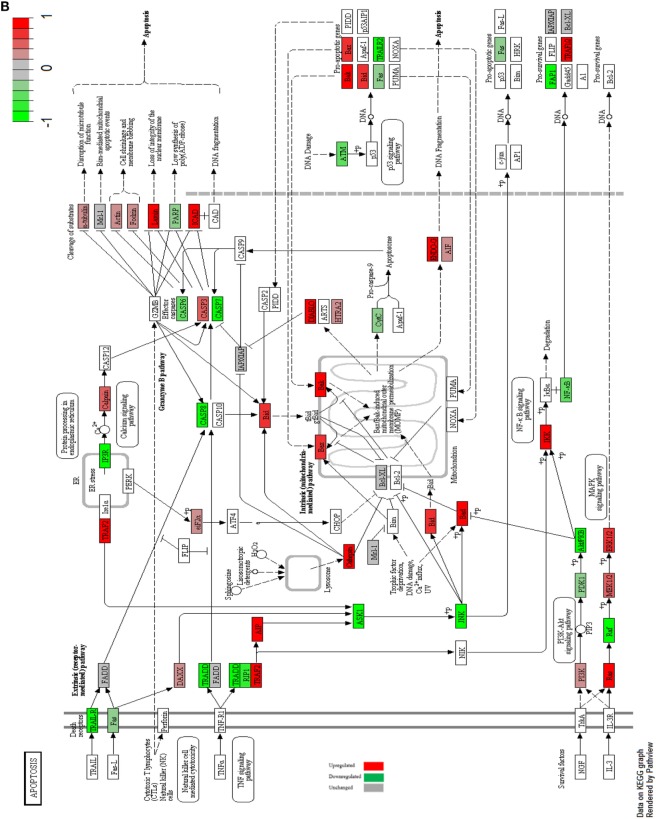
Apoptosis signaling in MCF-7 and MCF-10A. Apoptosis signaling in MCF-7 cells with highlighted up- and downregulated proteins after treatment with 10 mM metformin for 24 h in comparison with untreated control **(A)**. Apoptosis signaling in MCF-10A cells with highlighted up- and downregulated proteins after treatment with 10 mM metformin for 24 h in comparison with untreated control **(B)**.

Accumulating evidence has revealed an antitumorigenic effect of metformin on cell death. Apoptosis or programmed cell death is a conserved cellular activity among eukaryotes through the activation of caspases. Apoptosis is initiated (externally) through the activation of cell death receptors on cell membrane. Death receptor such as TNFR1, TRAILR, or CD95/Fas is activated *via* their specific ligands TNF-alpha, TRAIL, or Fas-L, respectively, resulting eventually in apoptosis (52). Metformin is associated with the downregulation of TNF signaling pathway *via* interfering with the activation of NF-kB by IKK in both MCF-7 and MCF-10A cell lines (53). The Downstream targets of the extrinsic pathway are downregulated in both MCF-7 and MCF-10A cells. The intrinsic pathway is activated independently of death receptor activation. Intrinsic pathway is activated in response to any cellular stress such as metabolic stress, DNA damage, ER stress, growth factor depletion, and ionizing irradiation. Upon the activation of intrinsic pathway, cytochrome c is released due to mitochondrial outer membrane permeabilization (MOMP), resulting eventually in apoptotic events *via* the effector caspases 3, 6, and 7 (52). In MCF-7 cells effector caspases were downregulated (Figure 5A), while in MCF-10A caspase 3 was upregulated (Figure 5B).

The cells final fait whether they will survive, proliferate, or undergo apoptosis is determined through the balance between the antiapoptotic and the proapoptotic proteins (52). Some proapoptotic proteins such as Bak and Bid were upregulated alongside with the downregulation of the prosurvival signals such as MCL-1, FAP1, and BCL in both cell lines with metformin induced apoptosis.

These results suggest that metformin induces the mitochondrial-mediated apoptotic pathway but not the receptor-mediated apoptotic pathway (54). In MCF-7 and MCF-10A mitochondrial-mediated secretion of DIABLO is upregulated which in turn inhibits the effect of IAP apoptosis inhibitory protein. The apoptosis inhibitory protein XIAP is downregulated.

The release of ENDO-G and AIF from the mitochondria was upregulated in both MCF-7 and MCF-10A cells. ENDO-G and AIF proteins are responsible for DNA fragmentation and subsequent apoptotic events independent of caspases (55, 56).

### Upregulated and Downregulated Proteins in MCF-7 Post Metformin Treatment

**Figure 6:**
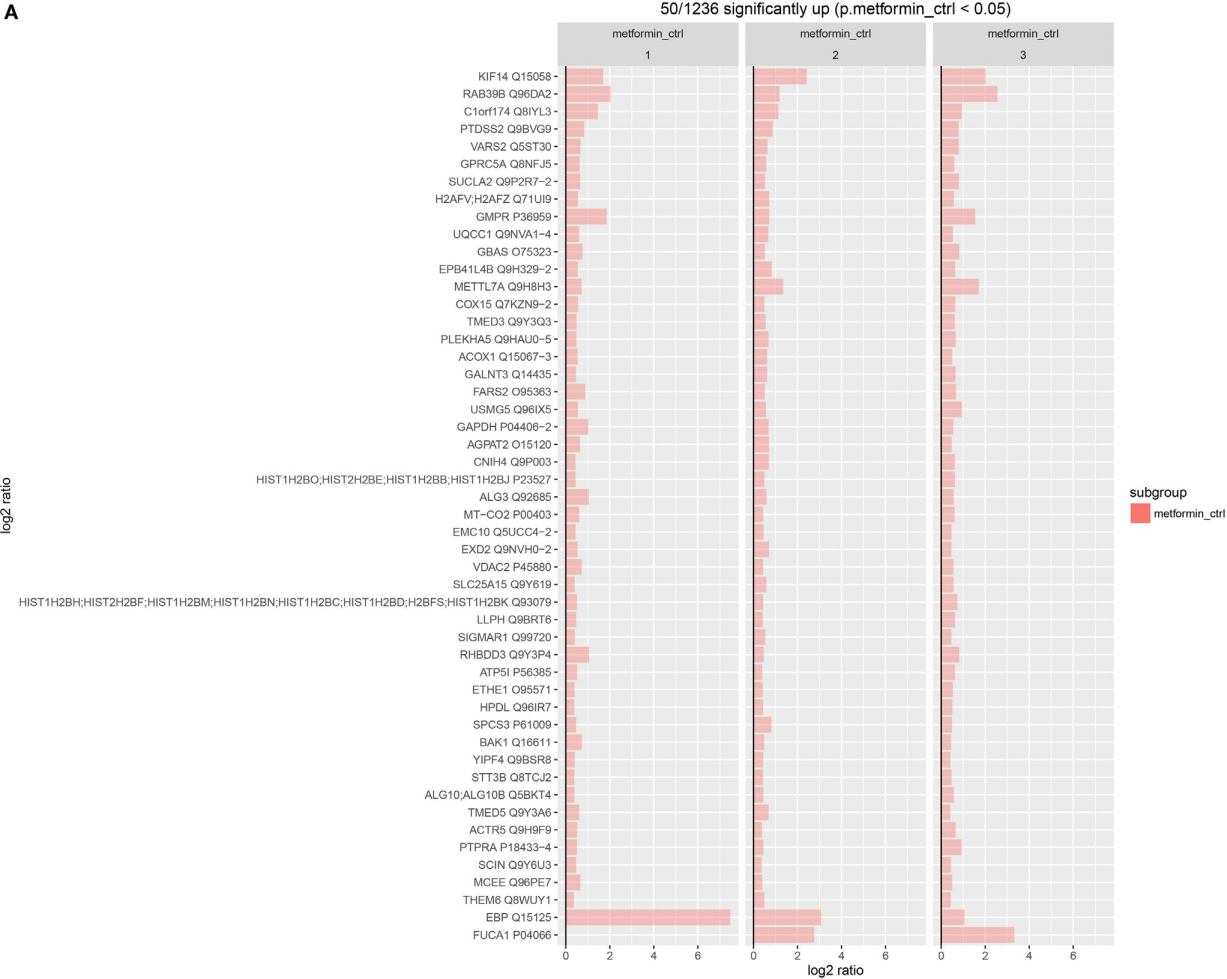

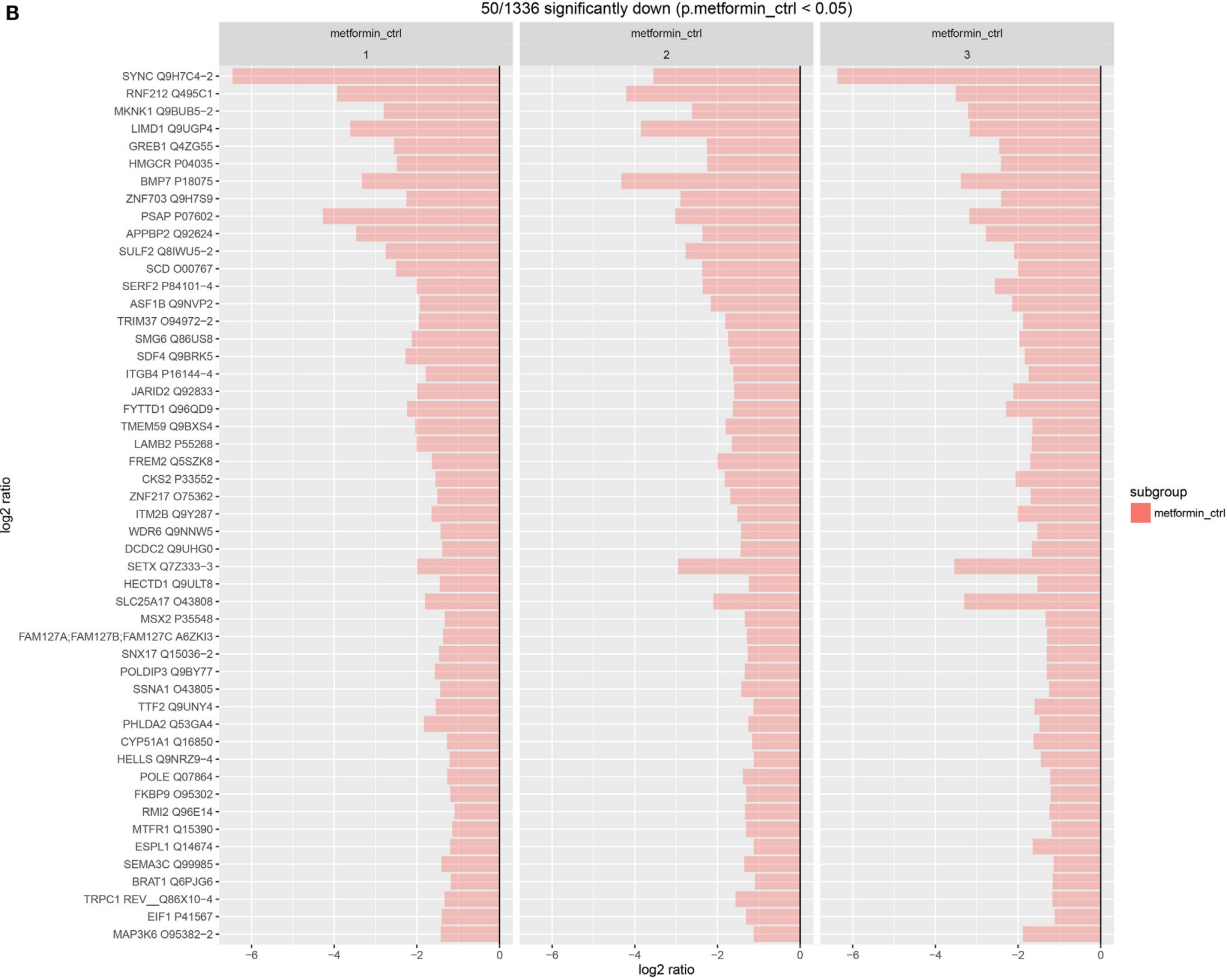
Statistical analysis for the significant upregulated and downregulated proteins. Statistical graph of the highly significant upregulated top 50 proteins in MCF-7-treated cells **(A)**. Statistical graph of the highly significant downregulated bottom 50 proteins in MCF-7-treated cells **(B)**.

We further studied the significant up- and downregulated proteins in metformin-treated MCF-7 to identify novel of molecular protein targets, which can be utilized in the future to develop a new therapeutic strategy for breast cancer. Statistically, we identified the top 50 highly significant upregulated proteins and the bottom 50 highly significant downregulated proteins. The resultant information was compared to UniProt and genecards databases. To better sort the data, we classified the proteins into three groups. First, genetic material and division processes. Second, cytoskeletal and membrane structural components. Third, vesicles regulation proteins. Among the upregulating proteins, we found that a number of them fall in the first group proteins such as VARS2, GPRC5A, H2AFV, GMPR, METTL7A, histones, and ACTR5 proteins. Some upregulated proteins also fall in the second group such as; PTDSS2, EPB41L4B, CNIH4, EMC10, VDAC2, and SIGMAR1 proteins. Other upregulated proteins in the third group such as; KIF14, RAB39B, TMED3, GAPDH, and TMED5 (Figure 6A). Of the highly significant upregulated proteins, some interesting proteins were found such as GBAS Glioblastoma amplified sequence, which is involved in mitochondrial oxidative phosphorylation (OXPHOS) thus it plays a role in energy metabolism (57). The mitochondrial ATP synthase subunit (ATP5I) is another upregulated protein that is involved in the formation of ATP synthase structure in the mitochondria and associated with the mitochondrial OXPHOS (46, 58), whereas the emopamil-binding protein (EBP) is an endoplasmic membranous protein that might be associated with pharmaceutical importance due to its high affinity to drugs in cells ER of different tissues (59, 60). FUCA1 tissue alpha-l-fucosidase is another upregulated in treated MCF-7 which is a lysosomal enzyme that degrades glycoproteins and glycolipids. High expression of FUCA1 was associated with the alteration of cell surface due to the fucosylation, which limits the invasiveness and metastasis of cancer cells in early stages. On the other hand, decreased expressions of this protein results in low prognosis and enhances malignancy progression (61, 62).

Among the downregulated proteins, we found a number of them falling in the first group such as; ASF1B, SMG6, JARID2, FYTTD1, ZNF217, CKS2, SETX, MSX2, POLDIP3, SSNA1, TTF2, HELLS, POLE, RMI2, ESPL1, and BRAT1 proteins. Some in the second group such as SYNC, LIMD1, HMGCR, PSAP, ITGB4, FREM2, LAMB2, ITM2B, and SLC25A17 proteins (Figure 6B). Among the interesting downregulated proteins found in treated MCF-7 is MAP kinase-interacting serine/threonine-protein kinase 1 (MKNK1), this protein may have a role in the response to cytokines and environmental stress, which may provide an effective strategy for cancer therapy (63, 64). Another downregulated protein is the growth regulation by estrogen in breast cancer 1 (GREB1). This protein is over expressed in ER + untreated (MCF-7) and can be associated with the signaling pathway of estrogen growth factor receptor that regulates cancer growth and proliferation, thus this protein at a great potential to be considered as a therapeutic target for breast cancer (65, 66).

## Discussion

Epidemiological studies elucidated that metformin decreases the incidence of morbidity and mortality rates of cancer in diabetic patients (9–11). Metformin was found to be associated with survival benefits in comparison with other antidiabetic drugs or in comparison with nondiabetic population (12). In cell culture, metformin has revealed growth inhibition effect and induced apoptosis in certain cancerous cell lines such as endometrial cancers, triple negative breast cancers, and glioma (23–25). However, the exact mechanism of which metformin exerts its beneficial effect on cancer is not well understood. This study employed cell cultures and protein extracts for proteomics analysis. We investigated the possible mechanisms of metformin’s effect on breast epithelial adenocarcinoma cells (MCF-7) in comparison with normal healthy cells (MCF-10A). This aimed to study protein targets resulted from metformin treatment in order to examine the possible signaling pathways, which may elucidate some explanations behind the beneficial effect of metformin on breast adenocarcinoma. Metformin was proposed to be an adjunctive medication to reduce the incidence and mortality in cancer patients of diabetic and non-diabetic status; for that reason, we thought to reduce the glucose concentration in the culture media, we cultured the breast adenocarcinoma MCF-7 cell line to mimic the conditions in cancer patients without diabetes. Glucose was reduced from approximately 24 mmol/L in DMEM media to 6 mmol/L in low glucose DMEM media which thought to be the common concentration in normal individuals. At first, we tested the viability of cells by flow cytometry for the cells cultured in low glucose media, and cells cultured in high glucose media to see the effect of glucose reduction on the viability of cells which showed no significant differences between both groups.

Some studies reported that high levels of glucose in culture media abrogate the effect of metformin on cancer (67–69). We examined the treatment by MTT assay in order to establish a dose response curve for different concentrations of the drug in 24 h. The cell viability was reduced dramatically to take a plateau trend starting at 7.5 mM, but we chose the 10 mM metformin with approximately 37% reduction in viability due to its frequent usage in other studies (27, 69–71). Cytotoxic effect on cancer cells were observed at concentrations ranging between 5 and 10 mM (70). The same concentration was applied to cells in different time points (2, 4, 6, 24, 48, and 72 h) and the viability was measured by trypan blue in 2, 4, and 6 h. There was no significant difference in comparison to the untreated control, while it was reduced to approximately 70% in 24 h and less than 25% in 24 and 72 h, which revealed that the appropriate time point for subsequent experiments was 24 h.

The performed apoptosis assay using flow cytometry revealed that the viability was reduced approximately to 27% in treated MCF-7 with 10 mM metformin for 24 h, which indicates that metformin induced apoptosis in MCF-7-treated cells. In contrast, it exerted a reduced cytotoxicity effect on MCF-10A normal breast epithelial cells, which reduced the viability up to 4% in comparison to the untreated MCF-10A cells. These results suggest that the effect of metformin on cancer cells exceeded its effect on normal cells and that metformin specifically targets the cancerous cells. In order to confirm metformin’s mediated induction of apoptosis in MCF-7 and MCF-10A cells, light microscopy images of untreated cells and 10 mM metformin-treated cells for 24 h were examined to view the morphology of apoptotic cell determined by blebbing and shrinkage of both cell lines. MCF-10A showed mild apoptotic appearance, which indicates that the effect of metformin on cancer cells exceeded the effect on normal cells. TUNEL assay was also performed to visualize the appearance of TUNEL-positive cells after treating MCF-7 cells with 10 mM metformin for 24 h, it showed a greater number of apoptotic TUNEL-positive cells in treated cells in comparison to the untreated control.

The cell cycle analysis revealed that metformin exerts its inhibitory effect on treated cell cycle by increasing the percentage of cells in G1 phase and reducing the percentage of mitotic cells comparing with the untreated controls, which indicate that metformin resulted in cell cycle arrest at G1 checkpoint. The difference in G1 phase percentage between the two cultures was approximately 7%, which is less than the difference reported in other studies (27, 71) due to the glucose reduction that lead to a state of energy deprivation and reduction in the building precursors needed for daughter cells according to Warburg’s hypothesis (72–74). Consistent with the antiproliferative effect of metformin in cancer cells, our data shows a decreased expression in many cell cycle kinases such as CDKs, CDC45, and CDC25C (44). On the other hand, MCF-10A-treated cells showed a decreased expression in CDC25C while CDKs retained some of their activities *via* CDK7, which was upregulated. We observed that a number of transcriptional factors activities were suppressed such as Rb the tumor suppressor protein (75), while Rb-related proteins p107 and p130 were upregulated. Thus, resulting in cell cycle arrest in G1 phase, however, the expression of Rb and its related proteins in MCF-10A cells remained unchanged after the treatment in comparison to the untreated control cells and with the presence of CDKs, Rb has been activated to repress E2F1 transcriptional activity. Other transcriptional factors such as SMAD2, SMAD3, and SMAD4 proteins were upregulated in MCF-7-treated cells by forming a transcription repressor complex that regulates cell proliferation, while this complex was interrupted in treated MCF-10A due to the downregulation of Smad2 and Smad3 proteins. DNA damage response in MCF-7 cells was initiated through several upregulated proteins such as ATM, ATR, and DNA-PK in a P53-dependent manner. In comparison, treated MCF-10A revealed a reduction of ATM and ATR expression, while DNA-PK remained unchanged when compared to untreated MCF-10A. The proteins responsible for chromosome maintenance and DNA repair in M phase such as Smc and Mad2 are upregulated in MCF-7-treated cells but downregulated in MCF-10A-treated cells. Our findings suggest that metformin exerts a higher magnitude effect on cancer cell cycle in comparison with normal breast epithelium cells.

The insulin receptor-signaling pathway is involved in cell growth, survival, and proliferation in response to various growth factors such as insulin. The downstream signaling of PI3K/Akt and Ras-MAPK pathways were downregulated in treated MCF-7 cells indicating that metformin reduces the survival and proliferation rate in cancer cells. mTOR was downregulated in MCF-7-treated cells which decreased the protein synthesis which was compensated by p70S6k upregulation. The downstream signaling of MAPK pathway was inhibited *via* the downregulation of MEK1/2 and ERK1/2 in MCF-7-treated cells (76) which made MEK 1/2 an attractive targets for therapies due to the potential of interrupting the MAPK signaling and consequently repressing ERK1/2 since they are the only targets of MEK1/2 (77, 78). The insulin receptor pathways were activated in MCF-10A cells after metformin treatment, indicating that metformin targets cancerous cells. It was observed that GLUT1 glucose transporter 1 was upregulated in both cell lines due to metformin treatment, which in return facilitates cellular glucose uptake. In one study of human fibroblast cells; the expression of GLUT1 was increased after treating cells with metformin indicating that nutrients deprivation stimulate GLUT1 vesicles assembly to the plasma membrane, where they allow glucose uptake into the cells (51).

Our data revealed that the extrinsic apoptotic signaling pathway is downregulated in both normal and cancer cells while the intrinsic signaling pathway was upregulated in both cell lines, suggesting that metformin initiates the intrinsic mitochondrial signaling pathway but not the extrinsic receptor signaling pathway (54). Furthermore, it was observed that effector caspases were downregulated posttreatment in MCF-7 cells while caspase 3 was upregulated in treated MCF-10A cells. On the other hand, ENDO-G and AIF enzymes were upregulated in both cell lines, suggesting that metformin induced intrinsic caspase independent apoptosis pathway in MCF-7 cells, while it induced both intrinsic caspase-dependent and -independent apoptosis pathways (55, 56). By analyzing the highly significant up- and downregulated proteins, we observed that some of the three groups of proteins; genetic material and division proteins, cytoskeletal and membrane structural components, and vesicles regulation proteins were deregulated. Deregulation of these proteins might shed light on this component as a metabolic-associated drug that exerts an effect of unknown mechanism on cancer. GBAS and ATP5I are two upregulated proteins that are associated with mitochondrial OXPHOS (45, 46, 57, 58). This observation suggests that the nutrients deprivation and energy depletion might be the cause of this increment in order to compensate through switching from pure glycolysis to (OXPHOS).

Emopamil-binding protein is another upregulated protein in MCF-7-treated cells. This protein is localized to the ER of different tissue cells and characterized with high affinity to drugs (45, 46, 59, 60). Increasing EBP by metformin might explain the ability of metformin to increase the efficiency of cancer treatments and to overcome chemotherapy resistance. Furthermore, cancer therapies could be tested against its affinity toward EBP for tailored therapeutic regimens of cancer. FUCA1 is an important protein involved in cancer progression. Metformin increased the expression of this protein resulting in alterations in cell surface fucosylation, thus limiting the invasiveness and metastasis of cancer cells (45, 46, 61, 62). These observations suggest that using this protein of importance in cancer therapy to inhibit disease progression, which could be achieved by prescribing metformin as a neoadjuvant drug. MKNK, a downregulated protein in treated MCF-7 cells, renders its effect on oncogenic transformation and cancer progression. This protein was found to be a potential therapeutic target in cancer treatment (45, 46, 63, 64). GREB1 a protein which is originally upregulated in ER + breast cancers such as MCF-7 cells and results in upregulation of ER survival signaling through the growth factor ligand, resulting in increased growth and proliferation (45, 46, 65, 66). Metformin treatment reduced GREB1’s expression, which might result with a negative progression of the disease, therefore GREB1 protein can be considered as another target of cancer therapy. In summary, this study revealed the effect of the biguanide metformin *in vitro* on cell survival, growth, and proliferation of cancer. Metformin was found to increase the apoptosis and decrease cell survival, growth, and proliferation in breast cancer cells with less cytotoxicity effect in normal breast cells. Overall, we can conclude that metformin treatment’s effect on cancer cells exceeds its effect on normal cells suggesting that it is a cancer-targeting agent. Among the highly significant up- and downregulated proteins, some of them are worth to be further studied such as; GBAS, ATP5I, EBP, FUCA1, MKNK1, and GREB1. Those proteins are implicated in cancer prognosis and at a great potential to be key targets for cancer therapy. Further studies are needed to examine the effect of metformin on the proteomic pathways against other types of cancers.

## Author Contributions

AM: design, performing experiments, revision, and submissions. LZ: performing experiments, design, data analysis, and writing. RR: revision.

## Conflict of Interest Statement

The authors declare that the research was conducted in the absence of any commercial or financial relationships that could be construed as a potential conflict of interest.
